# Protein and lipid content estimation in soybeans using Raman hyperspectral imaging

**DOI:** 10.3389/fpls.2023.1167139

**Published:** 2023-08-04

**Authors:** Rizkiana Aulia, Hanim Z. Amanah, Hongseok Lee, Moon S. Kim, Insuck Baek, Jianwei Qin, Byoung-Kwan Cho

**Affiliations:** ^1^ Department of Smart Agricultural System, Chungnam National University, Daejeon, Republic of Korea; ^2^ Department of Agricultural and Biosystem Engineering, Faculty of Agricultural Technology, Universitas Gadjah Mada, Yogyakarta, Indonesia; ^3^ National Institute of Crop Science, Rural Development Administration, Miryang, Republic of Korea; ^4^ Environmental Microbial and Food Safety Laboratory, Agricultural Research Service, United States Department of Agriculture, Beltsville, MD, United States; ^5^ Department of Biosystems Machinery Engineering, Chungnam National University, Daejeon, Republic of Korea

**Keywords:** hyperspectral Raman imaging, non-destructive measurement, spectral analysis, soybean, protein, lipid

## Abstract

Unlike standard chemical analysis methods involving time-consuming, labor-intensive, and invasive pretreatment procedures, Raman hyperspectral imaging (HSI) can rapidly and non-destructively detect components without professional supervision. Generally, the Kjeldahl methods and Soxhlet extraction are used to chemically determine the protein and lipid content of soybeans. This study is aimed at developing a high-performance model for estimating soybean protein and lipid content using a non-destructive Raman HSI. Partial least squares regression (PLSR) techniques were used to develop the model using a calibration model based on 70% spectral data, and the remaining 30% of the data were used for validation. The results indicate that the Raman HSI, combined with PLSR, resulted in a protein and lipid model *R_p_^2^* of 0.90 and 0.82 with Root Mean Squared Error Prediction (RMSEP) 1.27 and 0.79, respectively. Additionally, this study successfully used the Raman HSI approach to create a prediction image showing the distribution of the targeted components, and could predict protein and lipid based on a single seeds.

## 1 Introduction

Soybean, one of the most important crops globally, is also one of the best protein sources for animal feed and provides outstanding global food security (Philis et al., 2018). Additionally, soybean accounts for a significant share of the world’s oilseed production, accounting for up to 60% of the global demand (Egli and Crafts-Brandner, 2017). On dry-weight basis, mature raw soybean seeds typically contain between 35% and 40% proteins, 20% lipids, 9% dietary fiber, and approximately 8.5% moisture (He and Chen, 2013). The location, planting conditions, and variety affect soybean composition. Foods made from soy are excellent sources of vitamins, minerals, proteins, and fiber, and are low in saturated fat (Dukariya et al., 2020). In the past two decades, several studies have reported that regular soy consumption is linked to a comparatively lower incidence of various malignancies in nations that consume soy products (Liu, 1997). Moreover, soy protein has several benefits, such as lowering liver or blood triglycerides and increasing HDL cholesterol (Chao, 2008). All human cells and tissues require proteins to function, and all components of the essential body parts contain proteins (Leidy et al., 2015).

Since the past 30 years, the USA and Brazil have contributed to over 50% of the world’s soybean production (Pagano and Miransari, 2016). China and India produce 18.1 million and 9.3 million metric tonnes, respectively, accounting for more than 90% of the Asian output in 2019. However, differences exist between how soybeans are used in Asian and Western countries, which impact the soybean varieties grown in these regions. Asian nations primarily use soybeans to make tofu, soymilk, and other fermented cultural foods (Shin, 2011). Western nations, however, refine soybean for soybean meal or seed oil (Shin and Jeong, 2015). This consumption behavior requires the breeding-industry sector to specifically develop soybean seeds to meet market demand. The nutritional and functional properties of soybeans, such as proteins, oils, carbohydrates, and other minor components, can be modified by conventional seed breeding and genetic engineering.

To measure the chemical components of food and agricultural products, precise approaches such as the Kjeldahl and Soxhlet methods have become generally accepted (AOAC, 1990; Lopez-Bascon and de Castro, 2020). However, the work and time requirements of these methods make it challenging to economically assess the quality of the products. Additionally, using this technology to evaluate seeds in breeding programs is restricted by the destructive process and minimal weight need for extraction before analysis (Mæhre et al., 2018; Wang and Cheng, 2018). Therefore, as the modern agroindustry develops, there is a growing need for a quick and non-destructive method to predict the chemical components of a single seed in a large sample.

Raman spectroscopy is a non-destructive method for analyzing materials without damage. Raman spectroscopy leverages Raman scattering to provide detailed information about molecular vibrations, yielding high sensitivity for minor components with high accuracy and precision. In 1928, Raman discovered Raman scattering for the first time (Ferraro et al., 2003). When monochromatic light interacts with a sample, it experiences inelastic scattering, which can be used to determine the characteristics of the sample. When light strikes a substance, one of the following three things can occur: light may be absorbed, scattered, or indifferently interact with the substance. Raman spectroscopy enables “fingerprint identification” of chemical bonds and functional groups in molecules, and can reflect differences in chemical composition and molecular structure at the molecular level. The location, magnitude, and shape of the Raman peaks can reveal information regarding the molecular makeup or composition of the studied substances. The composition and its distribution of the target sample in spatial domain can be observed using Raman chemical imaging (Song et al., 2020). The Raman method has already been applied to predict proteins and lipids in soybean powder, and yields the best prediction performance (*R_p_^2^*) of over 0.90 and 0.80, respectively (Lee et al., 2013). It has also been demonstrated that utilizing seed samples and transmission Raman spectroscopy can accurately predict the bulk amounts of proteins and lipids in soybeans (Schulmerich et al., 2012; Singh et al., 2019). However, all previous studies that used pointer Raman are limited in their application to large samples, because each seed must be collected individually.

The hyperspectral imaging technology simultaneously captures spatial and spectral data, and it has been used to assess food and agricultural products. This method can simultaneously evaluate many seeds and identify the chemical compositions unevenly distributed within a single seed. Therefore, the hyperspectral imaging technique has developed into a potent tool for assessing the chemical makeup of seeds. Many previous studies have used hyperspectral imaging, especially NIR HSI, to detect, predict, and classify agricultural resources, for example, to determine starch content in a single kernel of corn seeds (Liu et al., 2020), and estimate oil composition and discriminate species of Brassicas seeds (da Silva Medeiros et al., 2022). However, the overlapping spectra of different compositions make it challenging to directly examine the chemical makeup of a seed using NIR hyperspectral imaging, though. The characterisation of chemical compositions is challenging because calibration procedures in chemometrics are required to interpret the spectra.

Raman hyperspectral technology simultaneously captures spatial and spectral data and has been used to assess food and agricultural products. Raman imaging is generally performed by gathering several spectra at specific locations on a sample. This yields data cubes containing the Raman signal strength assessed as a function of the x and y (spatial) and spectral dimensions (Cebeci-Maltaş et al., 2014). The “point-scan” method, which is based on the acquisition of hypercubes, records each point pixel-by-pixel. The capabilities of spectroscopy and machine vision are combined in a hyperspectral imaging system, enabling the concurrent acquisition of both internal-component spectral data and external picture data (Liu et al., 2022). The target-sample composition, distribution, and morphology can be observed using Raman chemical imaging combined with Raman spectroscopy and digital imaging. It has applications in several fields, including mineralogy, biomedicine, and threat detection (Qin et al., 2014). For example, it has been used to quantify benzoyl peroxide in flour (Qin et al., 2017), detect various adulterants in wheat flour (Lohumi et al., 2019), and inspect bacterial contamination in watermelon seeds (Lee et al., 2017). To the best of our knowledge, no research has been published on the use of Raman HSI for the non-destructive prediction of protein and lipid content in intact soybean seeds or to investigate the distribution of protein and lipid content throughout the seeds. In previous research, Raman hyperspectral imaging has been used to detect the compositions in maize seeds (Yang et al., 2018). However, that study only detected and characterized the distributions of protein and oil, and did not predict the chemical compositions of maize seeds. Therefore, this study aimed to examine the possibility of predicting the protein and lipid content in a single soybean seed using a Raman hyperspectral imaging system. Additionally, to ensure the accuracy of the seed-based performance model, a powder-based model for each of the targeted components was formulated to evaluate it side-by-side.

## 2 Materials and methods

### 2.1 Sample preparation

Rural Development and Administration (RDA), South Korea, supported the use of soybean seeds in this study. These samples were divided into two measurements: the first for the protein model, and the second for the lipid model. The samples for the protein model comprised three varieties that had already been classified into three classes from the RDA. The total number of samples for the protein model was 1491 seeds, and each variety was divided into a few groups, each containing seven seeds, including the (low-protein class) PI85089 variety (80 groups), the (medium-protein class) Shinhwa variety (80 groups), and the (high-protein class) Saedanbaek variety (53 groups). The total seeds of the lipid model were 5,790 soybeans comprising six varieties divided into a few groups that contained 30 seeds each, including PI85089 (30 groups), SLS90-101 (30 groups), Galmi (30 groups), Shinhwa (30 groups), Savoy (27 groups), and Saedanbaek (30 groups). Because of the minimum weight requirement of the Kjeldahl and Soxhlet methods for the reference value, the number of seeds in each group was determined in this manner.

### 2.2 Reference value

Protein and lipid contents were investigated using the Kjeldahl and Soxhlet methods. It was not possible to determine these compounds in a single seed because of the minimal weight required for sample extraction. Each soybean cultivar was ground using 1.6 grams (Kjeldahl method) and 4 g (Soxhlet method).

#### 2.2.1 Protein determination

Organic nitrogen was transformed into ammonium sulfate using the Kjeldahl method after digestion in strong sulfuric acid. A boric acid solution was prepared by distilling ammonia under alkaline conditions. The amount of nitrogen, indicating the amount of crude protein in the sample, was estimated by titrating the borate anions produced with standardized hydrochloric acid. The nitrogen content was then converted using a conversion factor which is 6.25, that has been used for almost all feed (Jiang et al., , 2014).

#### 2.2.2 Lipid determination

Using Soxhlet extraction, the maximum oil yield in the feed was determined by weighing 4 g of soybean powder in a cellulose thimble and placing it in a Soxhlet device. Extraction was performed for 10 h with 200 mL of hexane (63–65°C). Finally, the weight of the crude soybean oil was calculated using the maximum recoverable oil yield after removal of the solvent (Terigar et al., 2011).

### 2.3 Raman hyperspectral imaging

**Figure 1:**
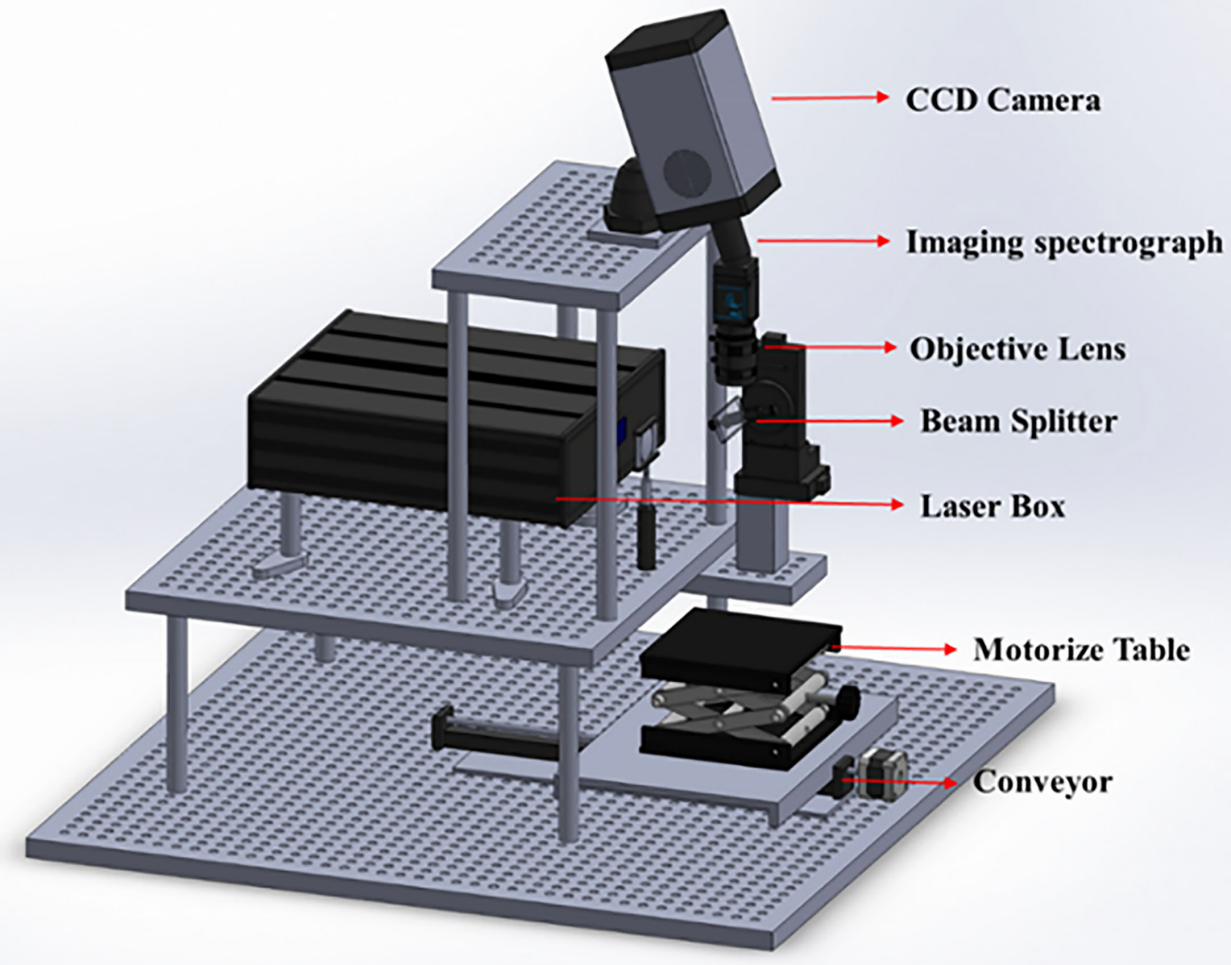
Schematic of the Raman hyperspectral imaging system.

Laboratory line-scan Raman hyperspectral imaging system was used in this research and is shown in **Figure 1**. The system was modified using several optical devices to provide uniform laser illumination of the sample and uniform Raman signal collection during sample data collection. First, a high-energy laser line was created by focusing the laser light from 19 emitters onto a 785-nm bandpass filter. Then, a cylindrical lens (f = 200 mm) was used to enlarge the relatively small laser line and achieve homogeneous intensity. The laser beam was then run via an engineering diffuser (EDI-L4100; Thorlabs, Hans Boekler, Dachau, Germany).

The resulting laser line was then projected onto a 785-nm dichroic beam splitter, which fixed its angle at 45° to project the laser line into the sample. The resulting Raman signal was projected onto a spectrometer using a slit in the dichroic mirror. When the resulting Raman signal passed through the filters, two long-pass filters were applied to remove any traces of the 785-nm laser line. A prism-grating-prism was also used to separate various wavelengths of light. Finally, a 16-bit CCD camera (iKon-M 934, Andor Technology, South Windsor, CT, USA) was positioned in the focal plane of the spectrometer to gather signals and produce images with an area array of 1024 × 1024 pixels. The produced image was uploaded to a computer and saved as a 3D hypercube in the ENVI format. **Figure 1** shows a schematic of line-scan Raman spectra.

A 100 × 100-mm black sample plate was filled with two groups of soybean seeds containing 60 seeds, which were then scanned using the described Raman hyperspectral imaging (HSI) method with a 0.2 mm step size. The system was turned on for 30 min before data collection to stabilize the laser sources and camera, enhance the spatial homogeneity, and reduce noise. The camera was then covered with an opaque cap while the laser was turned off to capture dark photos, which were later used to rectify the raw photographs. The generated Raman images were saved in the ENVI format as a 3D hypercube. The Raman signal was processed by polynomial fitting to exclude the fluorescence signals.

### 2.4 Fluorescence correction in Raman spectra

Raman spectra are frequently distorted by unfavorable background fluorescence noise from organic and biological samples. This fluorescent noise shifts the baseline and mutates the actual signal of the investigated sample. Additionally, the clarity and resolution of the spectra are also affected, which reduces the accuracy of detection of the target molecule, which is a difficulty in Raman spectroscopy (Qin et al., 2011). This problem can be solved by consistently fitting and correcting the baseline to precisely identify the necessary Raman peaks from the raw spectral data, reduce the fluorescence impact, and correct the baseline of the Raman spectra obtained in this investigation. Because of its effectiveness and simplicity, polynomial fitting was used in a recent study to eliminate the fluorescence background of Raman spectra (Lieber and Mahadevan-Jansen, 2003). Additionally, several previous studies have reported the use of various polynomial orders to study Raman spectra. For example, to examine lycopene in tomatoes, Qin et al. utilized an 8^th^-order polynomial equation, whereas Schulmeric et al. and Lee et al. employed 5th and 16^th^-order polynomial equations to remove the Raman spectra of soybeans (Qin et al., 2011; Schulmerich et al., 2012; Lee et al., 2013). In this study, we adopted 100 iterations and the 8^th^ polynomial order.

### 2.5 Preprocessing techniques

The initial spectrum data of the instrument may include significant noise owing to uncontrolled environmental factors and measurement-related variations. To improve the spectrum quality by removing unwanted fluctuations in the spectral data, these spectral data must be subjected to a proper mathematical analysis. This technique permits signal amplification, spectrum correction, and elimination of extraneous data such as background noise, path length fluctuation, scattering, and baseline shift (Rinnan, 2009). Normalization (mean, maximum, and range), multiplicative scatter correction (MSC), standard normal variate (SNV), and Savitzky–Golay derivative are the preprocessing techniques that can be used in spectroscopic data analysis (first and second). The selection of a specific preprocessing data analysis scheme is a significant challenge when utilizing vibrational spectroscopy techniques to examine the chemical composition of organic materials. Therefore, all of the mentioned preprocessing techniques were applied to the raw spectral data before analysis.

The most commonly used preprocessing method is mean normalization. The basic concept is to obtain the mean values from each dataset. In contrast, max and range normalization subtract the maximum or range data from each data point. The MSC approach involves fitting each spectrum to the average using least squares regression and calculating the preprocessed data by factoring in the slope and intercept of the regression. However, the preprocessed spectral data were calculated using the standard deviation of the SNV approach. A moving window approach was used to fit the data before Savitzky-Golay. The first derivative removed the baseline offset, creating a linear background at a constant level. In contrast, the baseline offset is typically removed using the second derivative to increase the spectral resolution and resolve close peaks.

### 2.6 Model development

The spectral data collected from all instruments were processed using several preprocessing techniques. First, protein and lipid contents were predicted using partial least squares regression (PLSR). This multivariate method, which can forecast the behavior of dependent variables based on sizable datasets of independent variables, combines multiple regression and feature-based extraction using the principal component analysis method. Spectral data (X) and response variables (Y) have a linear connection in the PLSR model, which makes it possible to forecast a component of the data variable.


(1) $$X=TP^{T}+E\;$$

(2) $$Y=UQ^{T}+F$$

(3) U=XB+G,

where X and Y represent spectra data and the protein and lipid content of soybeans. P and Q are loading matrices, and T and U are score matrices projected on linear combinations. The error matrices are represented by E, F, and G. The B matrix in Equation 3 contains regression coefficients.

The predicted root mean square error (RMSE) with the lowest value to determine the latent variables is represented by the following equation:


(4) $$RMSE=\sqrt{\frac{1}{z}\sum_{i=1}^{z}{\left(y_{i}-{\hat{y}}_{i}\right)}^{2}},$$

where z is the number of predictions, $\;y_{i}$ is the real reference value, ${\hat{y}}_{i}$ is the anticipated value from the PLSR, and the entire dataset is converted into calibration and validation datasets. The entire dataset was randomly assigned to 70% of the validation dataset and 30% of the calibration dataset. Finally, the model was constructed, encompassing the wavelength range of 900–1800 nm, after eliminating the poor intensity response above 1800 nm.

### 2.7 Prediction image

**Figure 2:**
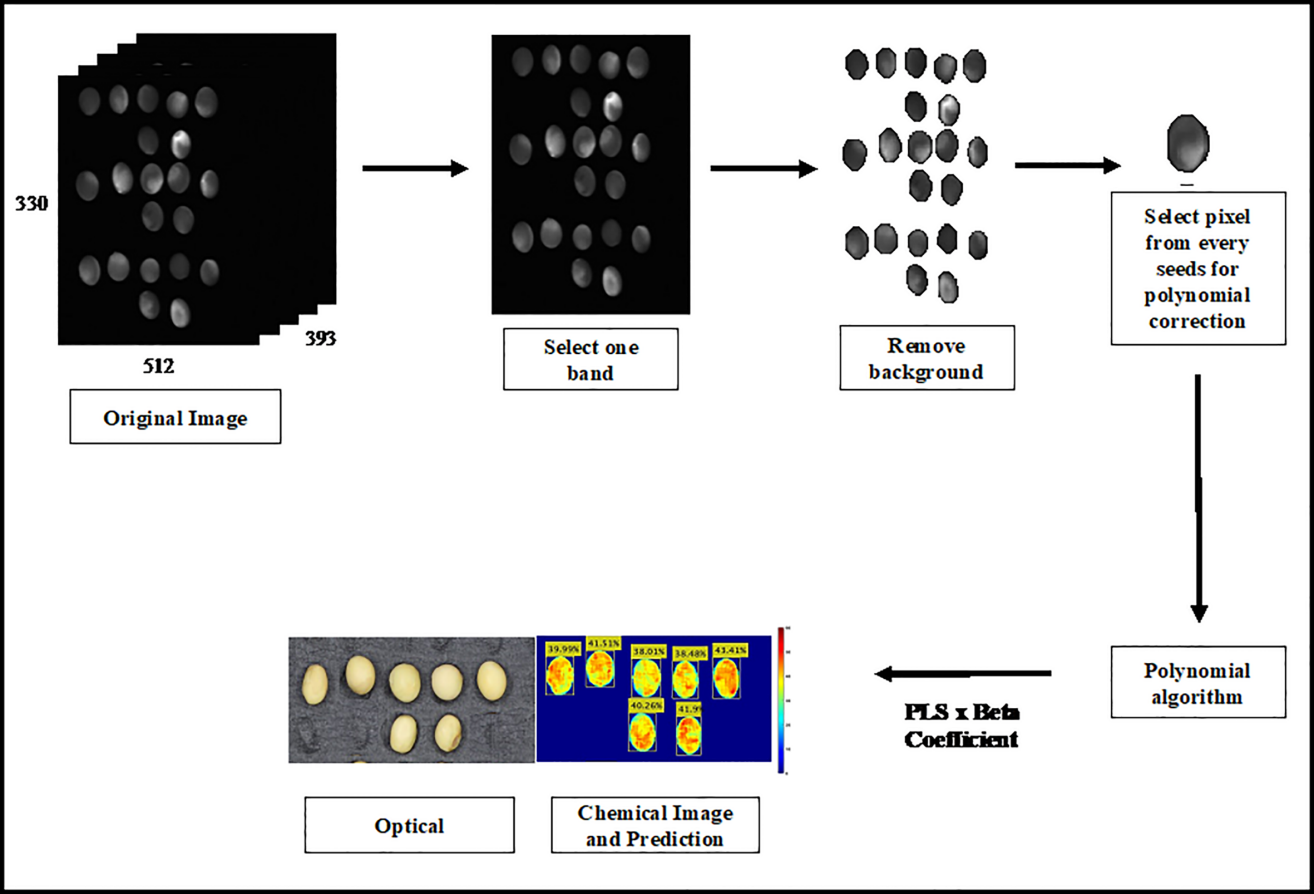
Strategy to generate the prediction image.

Hyperspectral imaging can generate a chemical image of the target chemical distribution of a sample. Therefore, chemical images were first created by multiplying the original HSI images with regression-coefficient vectors. However, the large size of the original Raman HSI image affected the iteration time required to generate the prediction image. The Raman original image contains 512 × 330 × 393 bands. The iteration time was reduced by selecting one band from the Raman image and removing the background. Polynomial correction pixels from all the band images were subsequently used to calculate the selected component concentration of the sample. Finally, the PLS was multiplied by the beta coefficient to generate the chemical images. The image processing process is outlined in **Figure 2**.

## 3 Results and discussions

### 3.1 Reference value analysis

Proteins and lipids in soybean seeds are essential for human health. Soybean is one of the cheaper protein sources in agricultural resources. In total, three varieties of the protein model and six varieties of the lipid model were used in this study to examine their chemical contents. **Table 1** presents statistical information about lipids and proteins, including the number of group varieties used in model development. Generally, the concentration range discovered in this investigation matched the conclusion offered by Wilson in that soybean has a protein range of 34.1–56.8 g/100 g (34.1–56.8%) of total seed weight (Wilson, 2004), and the lipid content ranges from 8.1% to 24% on the dry-seed basis (Medic et al., 2014).

**Table 1:** Reference values of protein and lipid content in soybean using the Kjeldahl and Soxhlet methods.

Sample	Mean ± SD	Maximum	Minimum	Number of soybean groups
Protein				
PI85089	34.1 ± 0.94	35.81	31.71	80
Shinhwa	38.81 ± 0.87	41.99	36.84	80
Saedanbaek	43.60 ± 1.56	45.77	38.37	53
Lipid				
PI85089	18.46 ± 0.70	19.51	16.87	35
Galmi	17.27 ± 0.54	18.57	16.14	35
SLS90-101	17.53 ± 0.65	18.74	16.27	35
Shinhwa	16.77 ± 0.52	17.82	15.47	35
Savoy	18.92 ± 0.80	20.2	17	27
Saedanbaek	13.87 ± 0.34	14.97	13.29	35

### 3.2 Model prediction of soybean protein and lipid content

**Figure 3:**
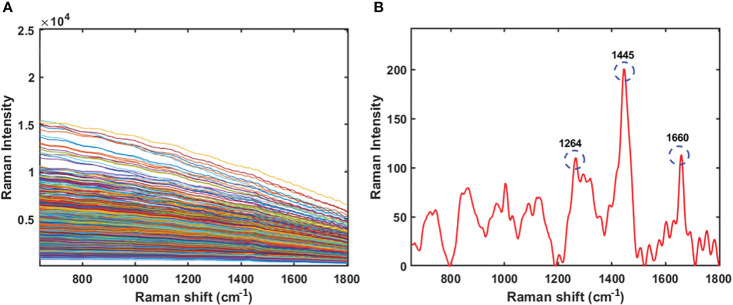
Original Raman spectra of soybean **(A)** and Raman spectra of soybean sample by removing the fluorescence signal using a polynomial equation **(B)**.

The original Raman spectra of the soybean samples are displayed in **Figure 3A**. Owing to the background fluorescence, there was a significant intensity variation. Therefore, the fluorescence signal from the original Raman signals of soybeans was subtracted using an 8th-order polynomial equation. **Figure 3B** shows identical spectra after removal of the fluorescence signal. These peaks represent different parts of soybean. The results of the PLSR model for predicting protein and lipid content using Raman hyperspectral imaging modified by removing the fluorescence signal are shown in **Tables 2** and **3**. The coefficient of determination value (R^2^) and standard error were used as evaluation metrics. The higher the correlation values, the better the model performance. Based on theseed and powder data for predicting protein content in soybean, the mean normalization preprocessing approach produced the best results: R_p_^2^ (0.90), RMSEP (1.27%), and 11 for the latent variable. By contrast, the powder result is R_p_^2^ (0.92), RMSEP (1.05%), and the lowest latent variable (LV) is 12. The lipid model results showed that the best preprocessing method for seed and powder samples using the SNV technique was the most effective preprocessing model. The R_p_^2^ results of the seed and powder samples are 0.82 and 0.84, respectively. Hence, according to Rinnan et al., mean normalization can be applied to spectral data, followed by SNV and MSC (Rinnan et al., 2009).

**Table 2:** Partial least squares regression model for predicting protein content in soybean using Raman hyperspectral imaging.

Preprocessing Method	R_c_^2^	RMSEC	R_p_^2^	RMSEP	LV
Seed Sample					
Mean normalization	0.95	0.84	0.90	1.27	11
Maximum normalization	0.95	0.87	0.90	1.30	13
Range normalization	0.95	0.87	0.90	1.31	13
MSC	0.95	0.82	0.88	1.41	12
SNV	0.95	0.85	0.89	1.34	11
SG first derivative	0.96	0.81	0.90	1.29	13
SG second derivative	0.96	0.82	0.79	1.87	16
Raw	0.95	0.86	0.90	1.28	12
Powder Sample					
Mean normalization	0.95	0.87	0.92	1.05	12
Maximum normalization	0.96	0.82	0.93	1.04	14
Range normalization	0.96	0.83	0.93	1.04	14
MSC	0.95	0.91	0.92	1.04	12
SNV	0.95	0.87	0.92	1.05	12
SG first derivative	0.94	0.99	0.88	1.33	14
SG second derivative	0.95	0.86	0.77	1.80	17
Raw	0.94	0.93	0.89	1.28	16

**Table 3:** Partial least squares regression model for predicting lipid content in soybean using Raman hyperspectral imaging.

Preprocessing Method	R_c_^2^	RMSEC	R_p_^2^	RMSEP	LV
Seed Sample					
Mean Normalization	0.88	0.58	0.82	0.80	11
Maximum Normalization	0.88	0.57	0.83	0.78	11
Range Normalization	0.88	0.58	0.82	0.78	11
MSC	0.88	0.58	0.79	0.86	11
SNV	0.88	0.59	0.82	0.79	9
SG first derivative	0.94	0.40	0.67	1.12	20
SG second derivative	0.48	1.21	0.23	1.78	8
Raw	0.84	0.66	0.73	0.98	11
Powder Sample					
Mean Normalization	0.86	0.64	0.84	0.69	11
Maximum Normalization	0.83	0.69	0.82	0.72	11
Range Normalization	0.83	0.70	0.82	0.72	11
MSC	0.87	0.62	0.82	0.72	12
SNV	0.87	0.60	0.84	0.70	11
SG first derivative	0.81	0.74	0.54	1.15	11
SG second derivative	0.27	1.45	0.11	1.58	5
Raw	0.81	0.74	0.77	0.82	12

The performance of the powder sample is better than that of the seed sample on the protein and lipid models, and it is denoted by the higher R_p_^2^ value of 0.92 of 0.84, respectively. This was because the powder sample was already ground and mixed into one sample, and most of the protein and lipid content was inside the bean. According to Kawamura, soybean seeds have the highest protein and lipid content in the cotyledon part, containing 43% protein and 23% lipid, while the seed coat only contains 9% protein and 1% lipid (Kawamura, 1967).Therefore, the macro- and micro-components were homogenized because the sample had been ground into sample powder.

**Figure 4:**
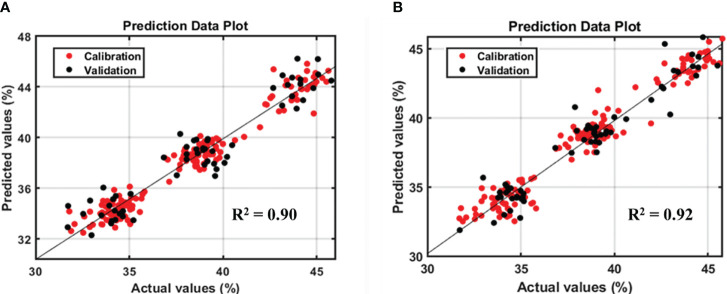
Prediction plot of protein model in soybean-seeds sample **(A)** and powder sample **(B)**.

**Figure 5:**
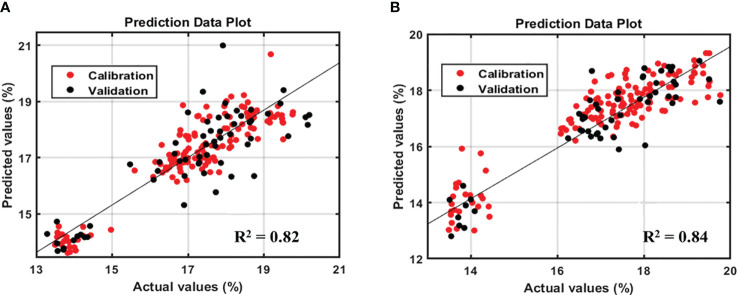
Prediction plot of lipid model in soybean seeds sample **(A)** and powder sample **(B)**.

However, based on the model performance, the protein model yielded better results than the lipid model. **Figures 4** and **5** show the prediction plots of the protein and lipid models, and differences can be observed between the two. The difference is that the protein model is already classified into the low, middle, and high classes by the soybean company, which could affect the performance value. By contrast, no lipid class was classified based on the data distribution of the lipid model. As reported by Hahn, if the measurements closely match the model predictions, the R^2^ result is high. When the R^2^ is low, the model’s predictions and the observations are significantly different, which indicates that several points were located outside the best-fit line (Hahn, 1973). In addition, according to the guidelines for the interpretation of R^2^ by William and Norris show that if a model has an R^2^ value greater than 0.8 then can be used with caution in most applications, including research whereas if R^2^ is more than 0.9 then the model can be used in most applications (Polinar et al., 2019).

**Figure 6:**
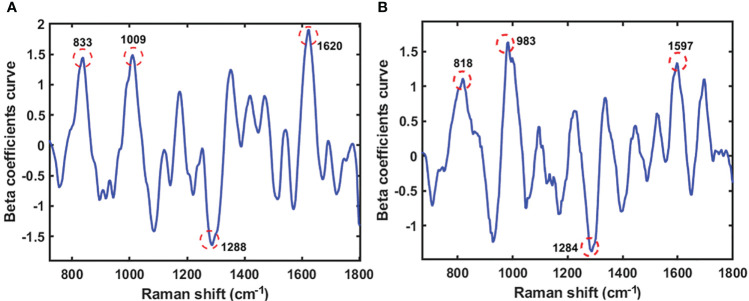
The beta coefficient curve of the PLS model of seeds sample **(A)** and powder sample **(B)** using Raman HSI techniques for predicting protein.

**Figure 7:**
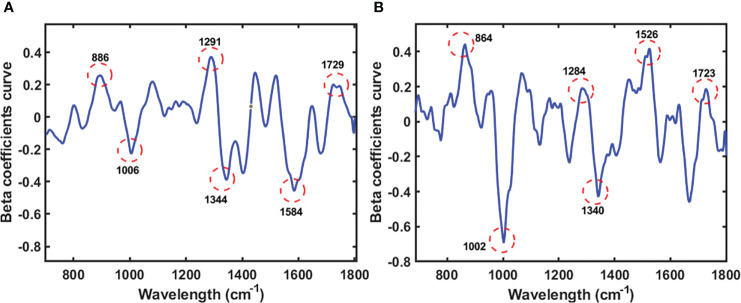
Beta coefficient curve of the PLS model of seeds sample **(A)** and powder sample **(B)** using Raman HSI techniques for predicting lipid.

Beta coefficients are crucial when analyzing multivariate data, because they quantify the number of units of standard deviation of the criterion variables in an equation involving multiple regression changes when a predictor variable’s standard deviation is changed while keeping the other predictor variables constant. The direction of association between the predictor variables (spectra) and substance variables was determined using the beta coefficient. The PLS model’s beta coefficient plots in **Figures 6** and **7** illustrate how the protein and lipid composition of various soybean varieties affect the energy absorbed by each variety. The peak in the graph indicates the result of the beta coefficient, which exhibits a similar pattern for both the seeds and powder. The first range is 800-833 cm^-1^ which indicates tyrosine doublet H-bonding. The second wave ranges from 983 to 1009 cm^-1^, representing glutamic acid and phenylalanine, which refer to an amino acid in soybean protein from a previous study (Kovalenko et al., 2006). Amide III, CH_2_, and CH_3_ deformations were observed between 1284 and 1288 cm^-1^. The last Raman peak at 1590-1680 cm^-1^ represents the protein content of C=N, which denotes a protein hand in histidine (Takeuchi, 2003). The moving atoms of the peptide backbone are represented by this compound, also known as amide I, as reported by Kurouski et al (Range et al., 2012).

Following the use of an 8^th^-order polynomial equation to eliminate the fluorescence signal, **Figure 7** shows the Raman spectra of soybeans for predicting lipid content. The band at approximately 860–890 cm^-1^ originated from C–O–C. Simultaneously, the bands in the ranges of 1002–1006 cm^-1^ and 1284–1291 cm^-1^ were attributed to C–C alicyclic and aliphatic chain vibrations, respectively, which are related to the lipid content, as reported in a previous study (Bai et al., 2022). The other peaks were between 1500 cm^-1^ and 1530 cm^-1^, corresponding to C=C. According to Lee (2017), the band associated with lipids was between 1680 and 1800 cm^-1^, specifically 1729 and 1723 cm^-1^, which were assigned to C=O. The chemical chain of C=O refers to fatty acids in lipid bonds (Lee et al., 2018).

### 3.3 Prediction image of protein and lipid content in soybean seeds and powder

**Figure 8:**
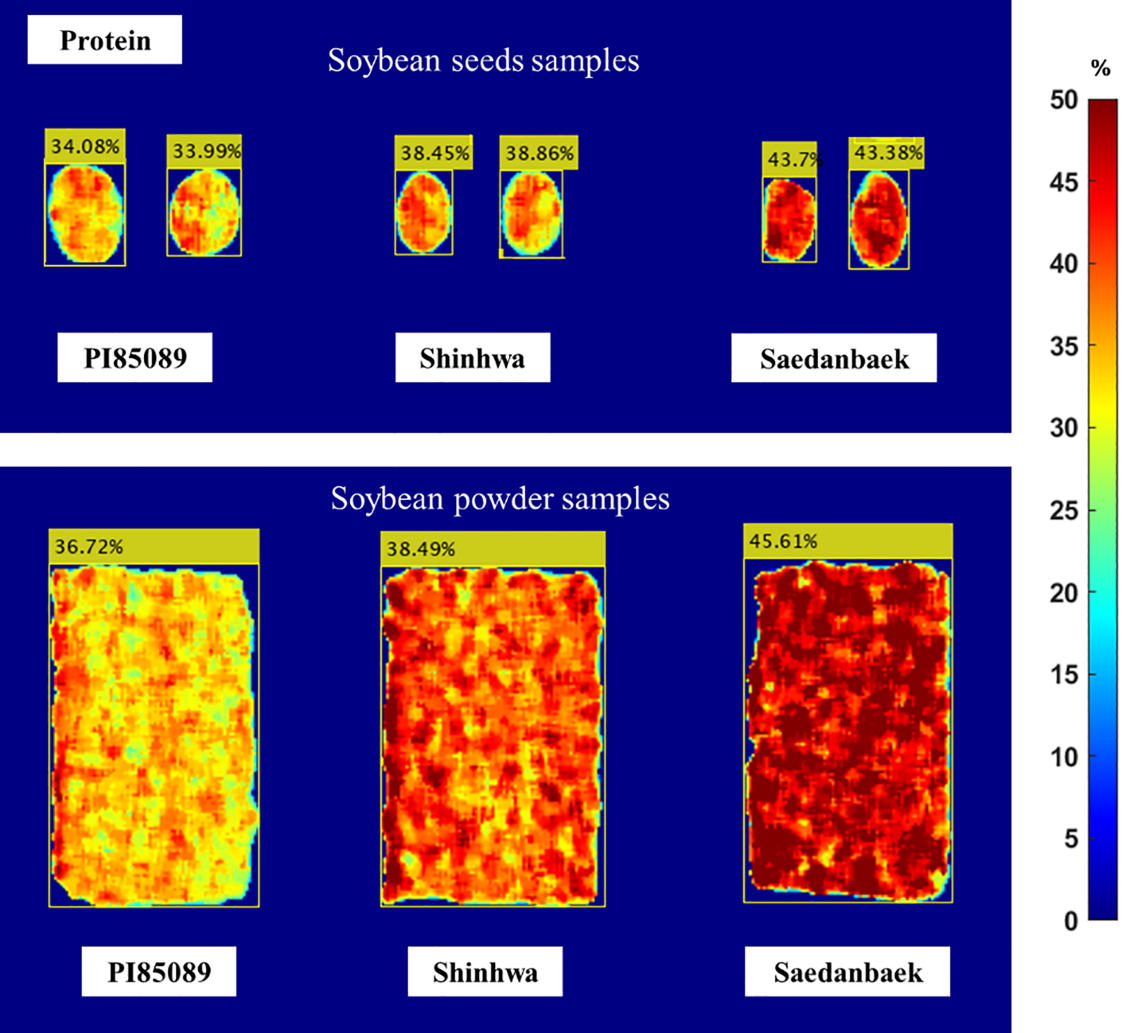
Prediction image of seed and powder samples constructed using Raman HSI for the protein content in soybean samples PI85089 (low class), Shinhwa (middle class), Saedanbaek (high class).

A prediction image was created to illustrate the distribution of proteins throughout the sample using data analysis results from the PLSR approach. **Figure 8** shows the protein distribution and prediction for each seed. The prediction-image results indicate that the variety PI85089 (low class) ranges from 31.71% to 35.81%, Shinhwa (middle class) from 36.84% to 41.95%, and Saedanbaek (high class) from 38.37% to 45.77%. The result of the prediction image shows a reasonable accuracy compared to the reference value. The PI82089 variety (low class) in the prediction image was 34.13% and 33.99%, whereas the mean protein in the reference value was 34.1%, indicating good performance. The second variety was Shinhwa, which was assigned to the middle-class protein predicted at 37.21% and 38.45%, whereas the reference value was 38.81%. The mean protein of the reference value for the Saedanbaek variety (high-protein class) was 43.60%, while the image predictions were 43.7% and 43.38%, respectively. The prediction imaging results demonstrate that the information shown is accurate, given the assigned reference value and protein class group.

**Figure 9:**
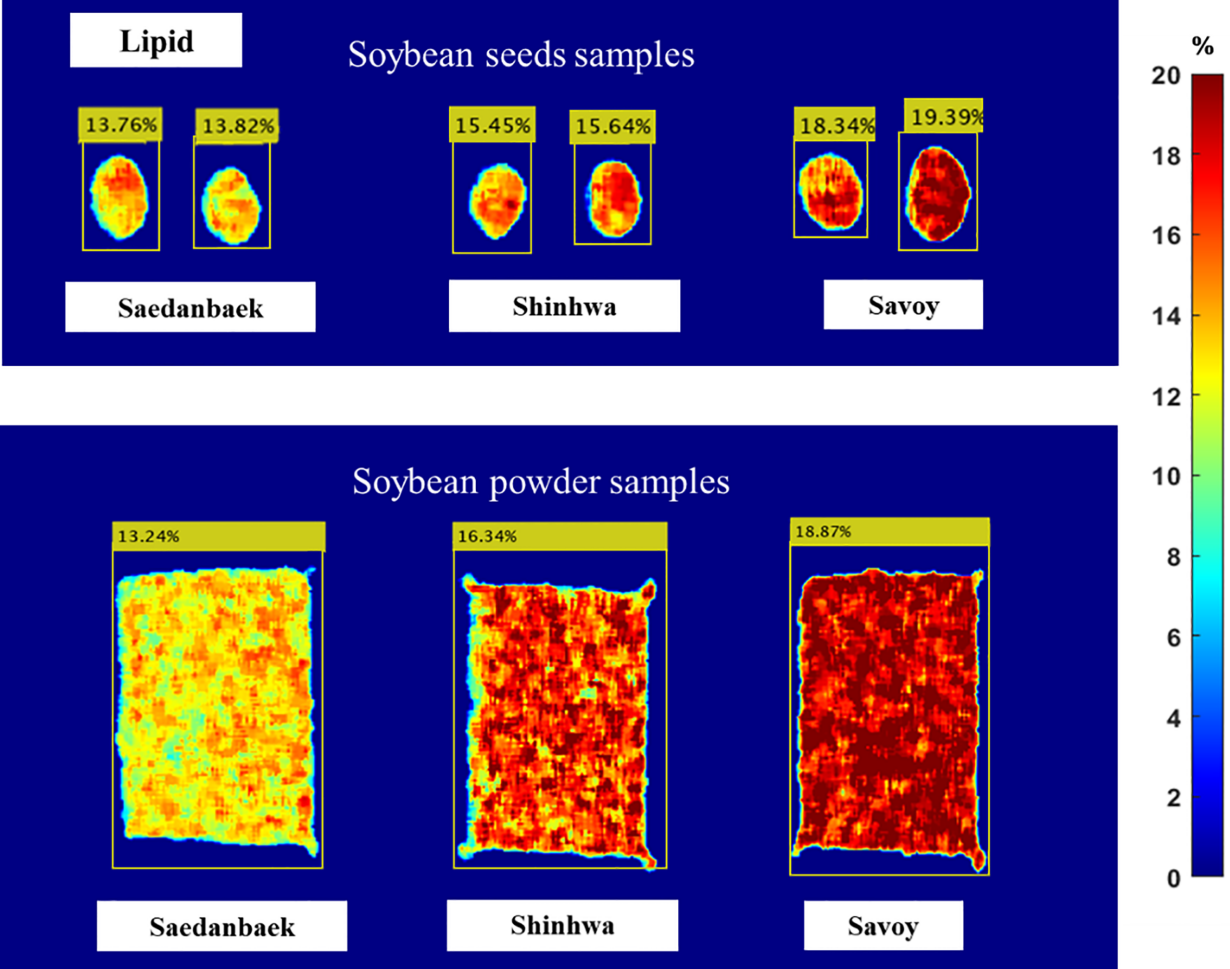
Prediction image of seed and powder samples constructed using Raman HSI technique for the lipid content in soybean sample: Saedanbaek, Savoy, and Shinhwa.

**Figure 9** shows the distribution of the lipid content in the soybean seeds in the prediction image. Analysis of the seed prediction image revealed that the lipid results were not as correctly predicted as the protein model. This was because the beta coefficient values from the preprocessing approach were necessary to create the prediction image. The R_p_^2^ value of the lipid model was 0.819, whereas the R_p_^2^ of the protein model was 0.90 for seed samples, indicating that the prediction model’s performance on lipid content was less accurate than the protein result, and affected the image prediction result. This is denoted by comparing the predicted image and lipid reference value obtained using the Soxhlet method. Although the result is not close to the mean result of the reference value, all results in the prediction image are in the reference value range, as shown in **Table 1**. For example, the Shinhwa prediction values were 15.45% and 15.64% while the mean result from the reference value was 16.77%. Nevertheless, the prediction result is still at the minimum to maximum value because the minimum value of the Shinhwa variety is 15.47%, and the maximum value is 17.82%.

As indicated by the corresponding color bars in **Figures 8** and **9**, blue represents low lipid and protein levels, and red represents high-level content. The red color on the surface of the powder, as illustrated in the figure, indicates that the protein content distribution spreads equally in the soybean powder. In contrast, the protein and lipid contents in intact seeds are only at a few points on the image of the seed. Therefore, although the image prediction represented the same protein content in both the seed and powder samples, the image prediction of the seed and powder samples was different in color distribution. Overall, unlike the human eye and conventional industrial color cameras, visual image prediction can show the distribution of chemical concentrations in samples. Additionally, the visualization results showed that HSI has several advantages over conventional spectroscopy in terms of the chemical composition and spatial contaminant identification of whole soybean seeds and powders.

## 4 Conclusion

Raman HSI methods were used to estimate the amount of proteins and lipids in soybeans using an entire seed sample. To examine the possibilities of these methodologies for measuring the chemical components of agricultural products, the possibility of predicting various types of targeted components was examined. We investigated the viability of employing a Raman HSI to predict the protein and lipid content in soybean seeds. The results indicated that Raman HSI combined with PLSR is a promising method for predicting protein and lipid content in a single soybean seed, presenting a performance prediction model (R_p_^2^) of 0.90 and 0.82, respectively. Additionally, the non-destructive technology demonstrated the estimation of protein- and lipid-content distribution in a single seed. The results demonstrated that the non-destructive measurement was consistent with the reference-value range for the representative varieties of soybean raised in Korea. Based on the capability of the chemical imaging of the Raman HSI for a single soybean seed, rapid seed sorting based on the protein and lipid content can be developed with the combination of an online or gravity assisted conveying system.

## Data availability statement

The original contributions presented in the study are included in the article/supplementary material. Further inquiries can be directed to the corresponding author.

## Author contributions

RA and B-KC conceived the overall contents and structure for this article. RA and HA led the data analysis drafted tables and figures. RA, HL, MK, IB and JQ acquired and analyzed sample information and conducted experiment. RA and B-KC reviewed successive drafts. All authors contributed to the article and approved the submitted version.
